# High sensitivity of gold nanoparticles co-doped with Gd_2_O_3_ mesoporous silica nanocomposite to nasopharyngeal carcinoma cells

**DOI:** 10.1038/srep34367

**Published:** 2016-10-03

**Authors:** Hui Wang, Songjin Zhang, Xiumei Tian, Chufeng Liu, Lei Zhang, Wenyong Hu, Yuanzhi Shao, Li Li

**Affiliations:** 1 State Key Laboratory of Optoelectronics Materials and Technologies, Sun Yat-sen University, Guangzhou 510275, China; 2 State Key Laboratory of Oncology in South China, Sun Yat-sen University CancerCentre, Guangzhou 510060, China; 3 Department of Biomedical Engineering, Guangzhou Medical College, Guangzhou 510182, China

## Abstract

Nanoprobes for combined optical and magnetic resonance imaging have tremendous potential in early cancer diagnosis. Gold nanoparticles (AuNPs) co-doped with Gd_2_O_3_ mesoporous silica nanocomposite (Au/Gd@MCM-41) can produce pronounced contrast enhancement for T1 weighted image in magnetic resonance imaging (MRI). Here, we show the remarkably high sensitivity of Au/Gd@MCM-41 to the human poorly differentiated nasopharyngeal carcinoma (NPC) cell line (CNE-2) using fluorescence lifetime imaging (FLIM). The upconversion luminescences from CNE-2 and the normal nasopharyngeal (NP) cells (NP69) after uptake of Au/Gd@MCM-41 show the characteristic of two-photon-induced-radiative recombination of the AuNPs. The presence of the Gd^3+^ ion induces a much shorter luminescence lifetime in CNE-2 cells. The interaction between AuNPs and Gd^3+^ ion clearly enhances the optical sensitivity of Au/Gd@MCM-41 to CNE-2. Furthermore, the difference in the autofluorescence between CNE-2 and NP69 cells can be efficiently demonstrated by the emission lifetimes of Au/Gd@MCM-41 through the Forster energy transfers from the endogenous fluorophores to AuNPs. The results suggest that Au/Gd@MCM-41 may impart high optical resolution for the FLIM imaging that differentiates normal and high-grade precancers.

Optical and magnetic nanomaterials have attracted increasing attention over the last few decades, owing to their dual modalities in optical and magnetic resonance imaging^1^,^2^,^3^. Gadolinium (Gd^3+^) is a paramagnetic relaxation agent used extensively in MRI owing to its ability to enhance the relaxation of neighbouring protons^4^,^5^,^6^,^7^. Co-doping of Gd^3+^ ions within the rigid matrices of MCM-41 mesoporous silica can effectively enhance their accessibility to water molecules and avoid release of its toxicity^1^,^8^. Gold nanoparticles (AuNPs) have been a common choice for contrast and therapeutic agents based on their superior optical properties, good biocompatibility and ease of bioconjugation with biomarkers to create nanosized contrast agents with molecular specificity^9^,^10^,^11^,^12^. Nanoprobes based on Gd^3+^ and AuNPs have exhibited significantly increased relaxivity values in MRI compared to those of chelated Gd^3+^ complexes^13^,^14^,^15^,^16^.

Nasopharyngeal carcinoma (NPC) is one of the most common cancers in Southeast Asia, with the highest incidence rates in the Guangdong province of China, Hong Kong, Malaysia, Indonesia, and Singapore, where NPC occurs 10–50 times more frequently than in Western countries^17^,^18^. Based on the 1978 WHO classification, all NPCs are histopathologically diagnosed as poorly differentiated squamous cell carcinomas^19^. Greater than 95% of NPCs are pathologically diagnosed as Type III undifferentiated carcinomas^18^, therefore, developing non-invasive methods of diagnosing the disease prior to tumour formation are urgently needed to improve patient survival rates. In this present work, the human poorly differentiated NPC cell line CNE-2 was chosen because its lack of differentiation is an important hallmark of mammalian malignancy and progression^20^. CNE-2 cells show very low IKKa expression^18^ and high expression of the epidermal growth factor receptor (EGFR)^19^, and are typically used as model cells in experimental and clinical research on NPC^20^. Our previous study revealed that Gd-incorporated MCM-41 mesoporous silica (Gd_2_O_3_@MCM-41) can efficiently enhance the MRI contrast and thereby improve T1-weighted images of the CNE-2 xenografted tumours in mice^21^.

Here, we report an investigation of the sensitivity of AuNPs co-doped with Gd_2_O_3_@MCM-41 (Au/Gd@MCM-41; Fig. 1) using the fluorescence lifetime imaging (FLIM) technique, in which the mean fluorescence lifetime of a chromophore is measured in each spatially resolvable element of a micrograph. The key feature for measuring the fluorescence lifetime of a chromophore is that lifetime directly depends on the excited-state reactions that are independent of excitation intensity, Chromophore concentration and light path length, all of which are difficult to control at the cellular level^22^. Therefore, the FLIM technique allows for exploration of the molecular environment of labelled macromolecules in the interior of cells and is ideally suited for early cancer diagnosis^23^,^24^. Lifetime images of both CNE-2 and NP69 cells were measured after excitation at wavelengths of 400 and 758 nm, respectively. The results demonstrate that Ga^3+^ has an important impact on the upconversion luminescence lifetime of AuNPs. The enhanced energy transfer from endogenous fluorophores to AuNPs may induce the longer fluorescence lifetime of AuNPs in CNE-2 after the uptake of Au/Gd@MCM-41 when compared with NP69 cells. The possible mechanism underlying the differences in FLIM images between CNE-2 and NP69 cells is discussed.

## Results

### Luminescence spectra and relaxations of Au/Gd@MCM-41

**Figure 2:**
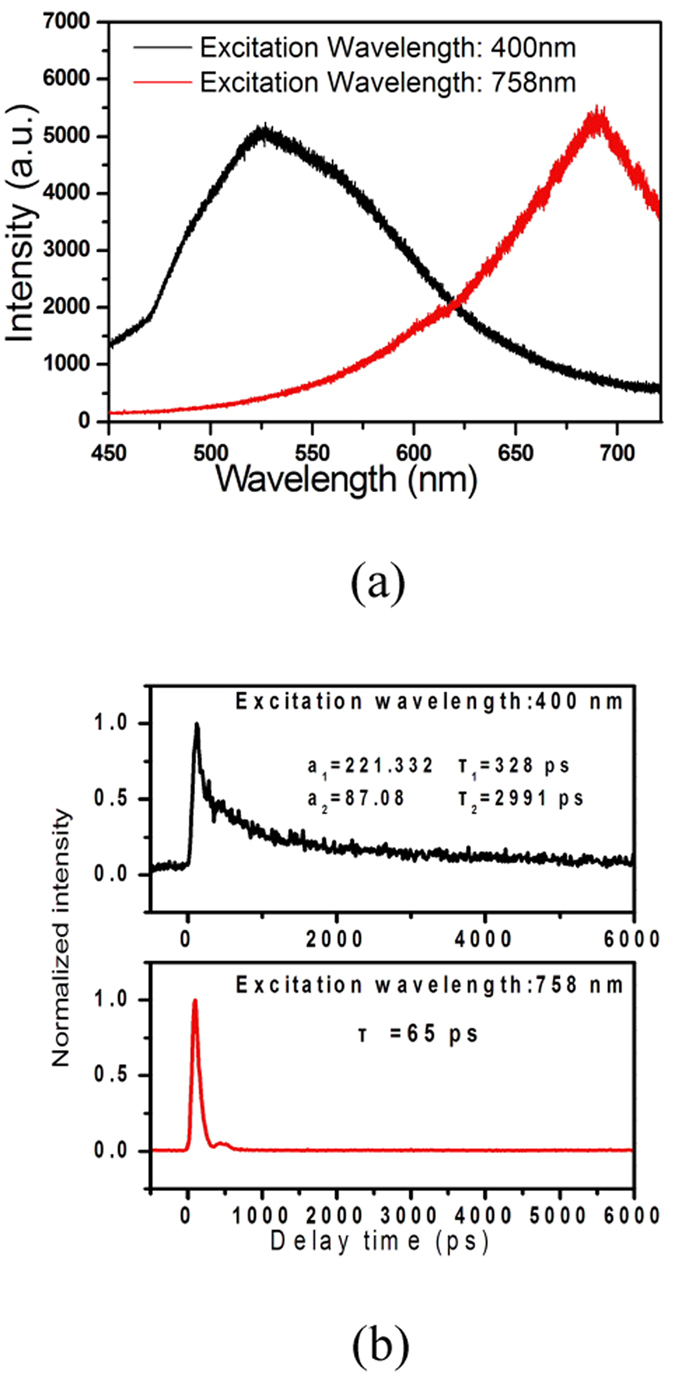
(**a**) Steady-state luminescence spectra of Au/Gd@MCM-41 excited at 400 and 758 nm. (**b**) Luminescence decay curves of Au/Gd@MCM-41 at the emission peaks of 525 and 688 nm.

As the optical probe, the absorption spectrum of AuNPs is characterized by an absorption band in the visible region at approximately 520 nm owing to surface plasmon resonance (SPR)^16^,^25^,^26^. For Au/Gd@MCM-41, the interaction between the unpaired electrons of Gd^3+^ and the SPR of the AuNPs significantly induced much stronger absorption and the appearance of a new absorption band around 749 nm (see Figs S1 and S2, Supporting information)^14^,^16^. The steady-state luminescence spectra of free Au/Gd@MCM-41 obtained with excitations at 400 and 758 nm are shown in Fig. 2(a). The emission spectrum with excitation at 400 nm exhibited a broad band with a peak around 525 nm, which is consistent with previously reported results and may be due to sp-electron-to-d-hole recombination of AuNPs with enhancement of the incoming and outgoing fields via plasmon resonances^27^,^28^. Under excitation at 758 nm, upconversion (UC) luminescence around 690 nm was observed, which likely stems from the two-photon excited interband photoluminescence of AuNPs^28^,^29^,^30^, as Raman scattering occurs at much longer wavelengths (see Fig. S3, Supporting information).

Figure 2(b) shows the time-resolved emissions of free Au/Gd@MCM-41 at the emission peaks of 525 and 690 nm (shown in Fig. 2(a)), which were well fitted using double and single exponential functions, respectively. The luminescence relaxation (λ_ex_ = 400 nm) was composes of fast (328 ps) initial and slow (2.99 ns) subsequent processes. The emission decay (λ_ex_ = 758 nm) was very fast, and the lifetime is approximately 65 ps.

### FLIM images with excitation at 400 nm

**Figure 3:**
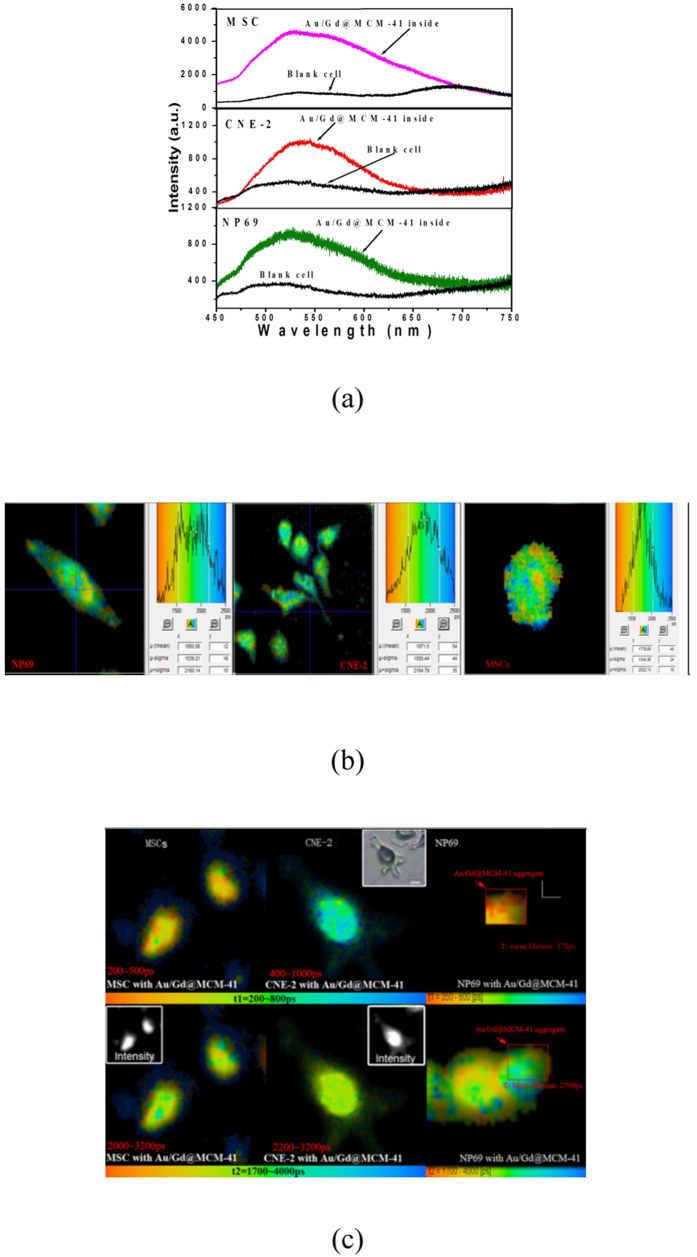
(**a**) Autofluorescence (black) and luminescence spectra (colours) after the uptake of Au/Gd@MCM-41 by MSC, CNE-2 and NP69 cells. The excitation wavelength was 400 nm. (**b**) FLIM autofluorescence images of NP69, CNE-2 and MSC cells (left to right). Excitation wavelength: 400 nm; emission peak: 525 nm. (**c**) FLIM images of MSC, CNE-2 and NP69 cells after absorbing Au/Gd@MCM-41 (excitation wavelength: 400 nm; emission wavelength: 525 nm). Top: FLIM images of τ_1_. Bottom: FLIM images of τ_2_. Insets: luminescence intensity images of MSC and CNE-2 cells.

The detection of autofluorescence (AF) allows the investigation of the epithelial layer, as the vast majority of nasopharyngeal tumours initiate in the epithelial tissue layer^17^. Many key endogenous fluorophores are specific to normal and cancerous NP cells and produce a strong autofluorescence (AF) signal under ultraviolet (UV) excitation between 260 and 400 nm^17^. Figure 3(a,b) show the AF spectra (black curves) and the FLIM images of AF from NP69 and CNE-2 cells, respectively, at 525 nm. As a control, mesenchymal stem cells (MSC) were also examined. The fluorescence decays in all 3 types of cells were well fitted by a mono-exponential function, and their lifetime distributions were similar (from 1.1 to 2.6 ns). Despite the AF lifetime imaging was performed on fixed cells in our experiment, the lifetime distributions in all 3 types of cells were consistent with previous results *in vivo* and in living cells^31^,^32^. Studies have shown that each endogenous fluorophore possesses its own emission band and wavelength maximum^33^. In our experiment, the 400 nm-excited lifetime images were obtained in the 525-nm channel, which have deviated from the emission peaks of the endogenous fluorophores with a high sensitivity to NPC^17^,^33^; such a deviation may have led to the absence of significant differences in AF lifetimes between normal (NP69 and MSC) and cancerous (CNE-2) cells^33^. The luminescence spectra (the colour curves) of CNE-2, NP69 and MSC cells after the uptake of Au/Gd@MCM-41 are also shown in Fig. 3(a) and are characterised by electron-d-hole recombination emission following the interband transition of the gold nanospheres^27^, and their emission intensity is much stronger than their AF^31^. Figure 3(c) shows FLIM images of CNE-2, NP69 and MSC cells under excitation at 400 nm after the uptake of Au/Gd@MCM-41. For NP69 cells, the fluorescence lifetime was analysed for the specific Au/Gd@MCM-41 aggregate areas. The lifetime data fit best with a bi-exponential function. The initial fast relaxation τ_1_ was followed by a slower process τ_2_. It appears reasonable to relate the fast lifetime component τ_1_ to the AuNPs in the Au/Gd@MCM-41. In this case, the mean lifetimes τ_1_ were 350 ps for MSC and NP69, 700 ps for CNE-2, which are somewhat longer than that of free Au/Gd@MCM-41 shown in Fig. 2(b). This lifetime lengthening can be attributed to Förster energy transfer (ET) from the endogenous fluorophores to the AuNPs^34^,^35^,^36^. Furthermore, the interaction between Gd^3+^ and the SPR of the gold nanospheres may effectively enhance the extinction coefficient of the AuNPs in the Au/Gd@MCM-41, as shown in Figs S1 and S2^16^, which can increase the overlap between the absorption spectrum of the AuNPs and the fluorescence spectra of the endogenous fluorophores^34^. Interestingly, the τ_1_ lifetime of the AuNPs in Au/Gd@MCM-41 becomes evidently longer in CNE-2 cells compared with the 2 types of normal cells. The lifetime of a fluorophore is dependent on a number of factors, including pH, oxygen levels, and refractive index. In particular, a low refractive index may hinder the relaxation of a fluorophore from its excited state and therefore prolong its lifetime^33^. Previous FLIM studies with endogenous fluorophores, such as nicotinamide adenine dinucleotide (NADH), in *in situ* breast carcinomas revealed that the tumour epithelium showed longer lifetimes than the normal epithelium^33^. On the other hand, a decreased refractive index may be capable of increasing the energy transfer efficiency from endogenous fluorophores to AuNPs^34^. Therefore, the longer τ_1_ lifetime observed in CNE-2 cells after the uptake of Au/Gd@MCM-41 is likely due to greater energy transfer efficiency. These results demonstrate the advantage of Au/Gd@MCM-41 for differentiating cancer cells (CNE-2) from normal cells (NP69), although determination of the absorption and emission peaks of endogenous fluorophores remains difficult because all cells contain numerous endogenous fluorophores. The FLIM images of the slow component τ_2_ should represent a mixture of the luminescence from the AuNPs in Au/Gd@MCM-41 and the autofluorescence of endogenous fluorophores. The τ_2_ lifetime distributions of the MSC and NP69 cells ranged from 2.0 to 3.2 ns, which is similar to those of CNE-2 cells, namely from 2.2 to 3.2 ns.

### FLIM images with excitation at 758 nm

**Figure 4:**
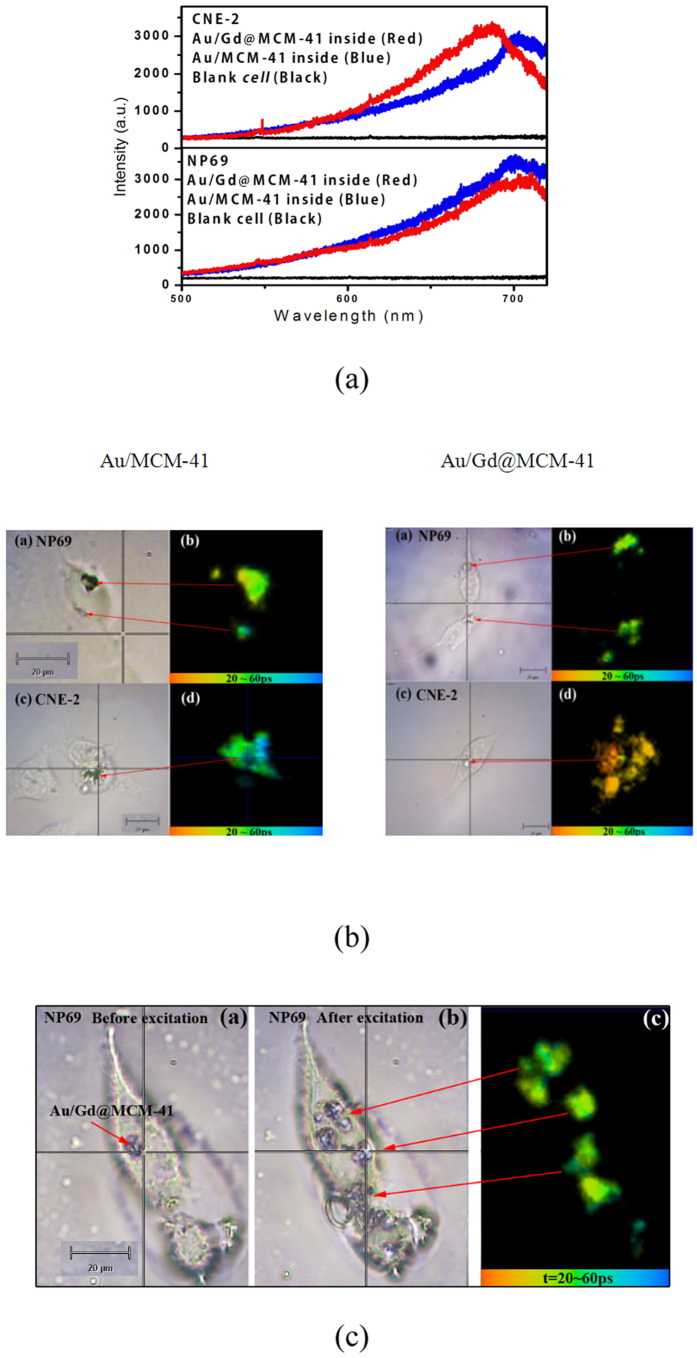
(**a**) Luminescence spectra of CNE-2 and NP69 cells excited at 758 nm after the uptake of Au/MCM-41 and Au/Gd@MCM-41. (**b**) Micrographs of NP69 (**a**) and CNE-2 (**c**) cells after the uptake of Au/MCM-41 (left) or Au/Gd@MCM-41 (right). FLIM images of NP69 (**b**) and CNE-2 (**d**) cells after the uptake of Au/MCM-41 (left) or Au/Gd@MCM-41 (right). Excitation wavelength: 758 nm; emission wavelength: 690 nm. (**c**) Au/Gd@MCM-41 nanocomposites aggregate in an NP69 cell after excitation at 758 nm. (**a**) Optical micrograph of an NP69 cell before excitation. (**b**) Optical micrograph of an NP69 cell after excitation. (**c**) FLIM image of Au/Gd@MCM-41 aggregation in an NP69 cell after excitation. Excitation wavelength: 758 nm; emission wavelength: 690 nm.

Multiphoton microscopy has gained significant popularity for biomedical imaging in recent years^31^,^37^. As a result of the longer excitation wavelength, multiphoton excitation microscopy offers increased penetration depth into living tissue and reduced sample photodamage^23^,^31^. In both CNE-2 and NP69 cells, 758-nm excitation can effectively avoid autofluorescence^17^, as confirmed by the control experimental result (the black curve) shown in Fig. 4(a). In this case, FLIM was then performed using Au/Gd@MCM-41 and non-Gd^3+^-doped Au/MCM-41 in living CNE-2 and NP69 cells. Figure 4(a) shows the upconversion luminescence spectra of Au/Gd@MCM-41 (red curves) and non-Gd^3+^-doped Au/MCM-41(blue curves) in CNE-2 and NP69 cells. The spectral profiles are consistent with the two-photon-induced luminescence of AuNPs^29^,^30^. The presence of the Gd^3+^ ions resulted in a slight blue shift of the emission peak in CNE-2 cells; however, the difference cannot be distinguished with conventional intensity-based microscopic techniques. The FLIM images of Au/MCM-41 and Au/Gd@MCM-41 in NP69 and CNE-2 cells are shown in Fig. 4(b). The lifetime data fit best to a mono-exponential function. The lifetime of two-photon-induced-luminescence of AuNPs was significantly shortened in cells. For Au/MCM-41, the lifetime distributions in CNE-2 and NP69 cells were similar (30 to 60 ps), and the mean lifetime was 45 ps. However, CNE-2 cells exhibited a much shorter lifetime (approximately 27 ps) than NP69 cells (~40 ps) with Au/Gd@MCM-41. Thus, the introduction of Gd^3+^ ions into the nanoprobe is clearly responsible for the shorter lifetime in CNE-2 cells.

## Discussion

The relaxation of a water proton from a spin state with high energy to equilibrium following the radiofrequency pulse is an important determinant of magnetic image contrast in MRI^5^,^6^,^7^. The relaxation time (T1) of a biological sample reflects the physical and chemical properties in the environment surrounding excited water protons. The paramagnetic ion Gd^3+^, which is presently used in clinical medicine as a contrast agent, possesses unpaired electrons, which may be highly sensitive to diseased tissue^6^,^14^,^21^. This sensitivity may administrate its dipole-dipole interaction with water protons and effectively shorten the spin-lattice relaxation time of the diseased cells, especially cancer cells^6^,^14^,^21^. SPR is an intrinsic feature present in AuNPs. Previous studies have demonstrated that the surface plasmon enhanced magneto-optical (MO) interaction in composite magnetic/plasmonic nanosystems^38^. Gadolinium-gold nanohybrids have exhibited enhanced contrast for MRI^13^,^14^,^15^,^16^. Such enhanced contrast may be attributed to plasmon-enhanced MO effects, which contribute to the relaxivity of bulk water molecules in addition to the contribution from the electron spin of the Gd^3+^ ions^15^,^38^. It has been demonstrated that the multiphoton-absorption-induced luminescence (MAIL) from gold nanoparticles over a broad range of dimensions is efficiently generated via near-infrared excitation^25^. The visible emission of Au/Gd@MCM-41 at approximately 688 nm, as shown in Fig. 2(a), may be explained by the two-photon-absorption interband transitions of the d-band electrons into the conduction band and the subsequent radiative recombination^28^. Previous studies have reported that MAIL from gold nanostructures is strongly influenced by the local surface plasmon effect^28^,^39^, which can enhance all of the radiative and nonradiative properties of AuNPs. For Au/Gd@MCM-41, our experiments using the X-ray photoelectron spectroscopy (XPS) confirmed the interaction between Gd^3+^ ions and AuNPs^16^. The efficient MO coupling through the surface plasmon effect of AuNPs may enhance surface plasmon oscillation and the nonradiative decay of excited AuNPs^30^; therefore, a shorter luminescence lifetime was observed in CNE-2 cells, as shown in Fig. 4(b). This case resembles the Gd^3+^-enhanced relaxivity of water protons in MRI. On the other hand, aggregates of gold nanoparticles can lead to field enhancement owing to plasmon resonance via near-infrared excitation^37^. Such aggregate-enhanced two-photon autofluorescence has been observed in fixed and live Chinese hamster ovary (CHO) cells and in NIH3T3 rat kidney epithelial cells^37^. In our experiment, Au/Gd@MCM-41 aggregates with diameters of approximately 130 nm were clearly visible, as shown in Fig. 4(b), with 758-nm excitation. The aggregation become more significant after excitation, as shown in Fig. 4(b,c). Therefore, the MO effect may be further enhanced through AuNP aggregate formation, which may contribute to the enhancement of nonradiative relaxation in CNE-2 cells. In addition, the MO effect may induce a new near-infrared absorption band (see Fig. S2, supporting information), which will be identified in future study.

## Conclusions

We investigated the optical sensitivity of a novel magnetic and optical nanoprobe (Au/Gd@MCM-41) to nasopharyngeal carcinoma cells (CNE-2) by FLIM. The AuNPs in Au/Gd@MCM-41 were responsible for the observed photoluminescence in both CNE-2 and NP69 cells when excited at 400 or 758 nm. Surface plasmon oscillation plays an important role in the luminescence relaxation processes of the AuNPs. The surface plasmon effect enhances the interaction between Gd^3+^ and the excited AuNPs, which results in more rapid relaxation of two-photon luminescence in CNE-2 cells. The difference in the AF between cancer and normal cells can be demonstrated by the Förster energy transfer from the excited endogenous fluorophores to the AuNP surface. Au/Gd@MCM-41 imparts much greater resolution (on a picosecond time scale) to optical imaging than to MRI, which has a resolution on the order of a millisecond. In summary, owing to its excellent optical sensitivity to CNE-2 cells and its nontoxicity, Au/Gd@MCM-41 may be an ideal diagnostic probe for early nasopharyngeal carcinoma. *In vivo* experiments with additional cancer cell lines are underway.

## Methods

### Synthesis and characterisation of Au/Gd@MCM-41

The synthetic strategy followed a protocol similar to that described previously^8^,^16^,^40^.

### Cell lines and culture conditions

MSC (mesenchymal stem cells) were cultured in DMEM/F-12 (Dulbecco’s modified Eagle’s medium/F12, Gibco (Life Technologies, Gaithersburg, MD, USA) supplemented with 10% FBS (fetal bovine serum, Invitrogen, Invitrogen, Carlsbad, CA). NP69 (normal nasopharyngeal epithelium) cells were incubated in keratinocyte serum-free medium (K-SFM, Gibco) with bovine pituitary extract (BPE, 0.05 mg/mL, Gibco) and epidermal growth factor (EGF, 0.005 μg/mL, Gibco). CNE-2 (human nasopharyngeal carcinoma) cells were maintained in RPMI 1640 media (Gibco) with 10% FBS. The cells were incubated at 37 °C with 5% CO2. Cells in logarithmic growth phase were digested using 0.25% trypsin (Gibco). Approximately 500–1,000 cells were seeded into confocal dishes and incubated for 12–24 h. The cells were then washed 3 times with PBS (phosphate-buffered saline) to remove suspended cells before being incubated with the dual-modality nanoprobes (100 μmol/L). The culture medium was again removed 12 h after co-incubation, and the cells were washed 3 times with PBS to remove the remaining Au/Gd@MCM-41 and dead cells.

### Fixed cells with Au/Gd@MCM-41 and blank control cells for FLIM

The samples that were studied at an excitation wavelength of 400 nm were fixed using 0.5% glutaraldehyde and 4% paraformaldehyde for 15 min at room temperature before measurement. In addition, blank control cells without Au/Gd@MCM-41 were used to evaluate the autofluorescence of the cells.

### Live cells with Au/Gd@MCM-41 and blank control cells for FLIM

Living cells were examined with 758-nm excitation, and blank control cells without Au/Gd@MCM-41 were used to evaluate the autofluorescence of cells.

### FLIM spectroscopy

FLIM was performed using a Renishaw confocal imaging spectrometer system (inVia Reflex, UK). A XY piezo stage scanning system (consisting of a controller and an amplifier for operation with a GVD-120 and a P-527.2CL stage, Becker and Hickl) was used to acquire high spatial resolution. The excitation source was a Ti-sapphire laser (tuning range of 690–1040 nm, Mai Tai, Spectra-Physics, USA), which generates pulse widths of approximately 100 fs at a repetition rate of 80 MHz and a pulse energy of 2 nJ. The second harmonic 400- and 758-nm (10-nm band width) pulses were used as the excitation light. The fluorescence from each pixel was detected using a MCP-PMT detector (SPC-150, HAM-R3809U-50, Hamamatsu, Japan) and fed into a TCSPC card (HRT-41, Becker and Hickl) to record the fluorescence lifetime. Furthermore, a reference signal was also fed into the TCSPC card for comparison with the electronic signals, which were used to define the time of signal arrival. The system response was faster than 30 ps^41^. The FLIM data were collected for 30-60 s, and fluorescence lifetime data were imported into data analysis software for FLIM (SPC-Image, v.3.2 Becker and Hickl), where a Levenberg–Marquardt routine for non-linear fitting was used to fit the fluorescence decay curve collected for each pixel in the 256 × 256 pixel array; the data were subsequently displayed using colour-coded images^33^. Corrections for noise were made using a calibration of the instrument response function. Herein, the data fit best with single- or double-exponential functions.

## Additional Information

**How to cite this article**: Wang, H. *et al*. High sensitivity of gold nanoparticles co-doped with Gd_2_O_3_ mesoporous silica nanocomposite to nasopharyngeal carcinoma cells. *Sci. Rep.* **6**, 34367; doi: 10.1038/srep34367 (2016).

## Supplementary Material

Supplementary Information

## Figures and Tables

**Figure 1:**
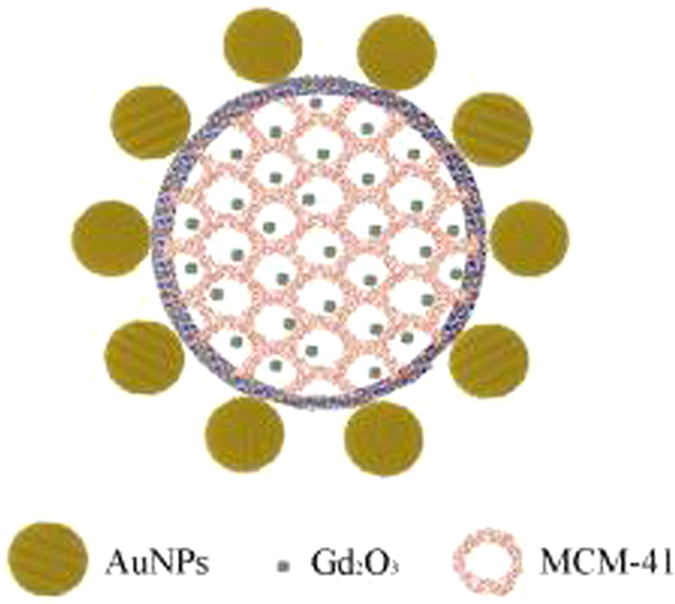
Optimised structure of an Au/Gd@MCM-41 particle with a diameter of approximately 100 nm. The size of the Gd_2_O_3_ cluster is approximately 1 nm. The pore size is approximately 2.9 nm. The average diameter of the gold nanospheres is approximately 30 nm.
