# Supplementary material for: High sensitivity of gold nanoparticles co-doped with Gd2O3 mesoporous silica nanocomposite to nasopharyngeal carcinoma cells
Source: Sci Rep. 2016 Oct 3;6:34367. doi: 10.1038/srep34367 (PMC5046069; doi:10.1038/srep34367)
Supplement: Supplementary Information [file srep34367-s1.pdf]

High sensitivity of gadolinium and colloidal gold nanocomposite in nasopharyngeal carcinoma cell

Hui Wang,[*,†] Songjin Zhang,[†] Xiumei Tian,[§,‡] Chufeng Liu,[†,‡] Lili Wang[†], Lei Zhang,[†] Wenyong Hu,[†] Yuanzhi Shao,[†] and Li Li[*,‡]

1. The steady-state absorption spectra were performed on a ultraviolet (UV)-visible absorption spectrophotometry (UV-3150, Shimadzu Scientific Instruments, Inc., Japan).

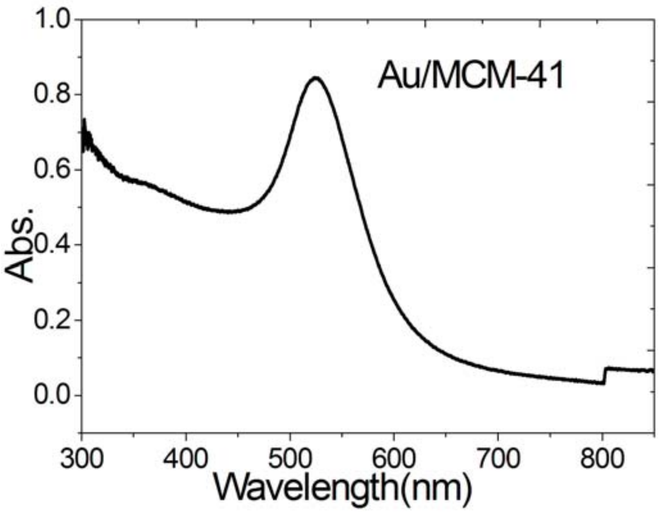

Figure S1 the steady- state absorption absorption spectrum of Au/MCM-41.

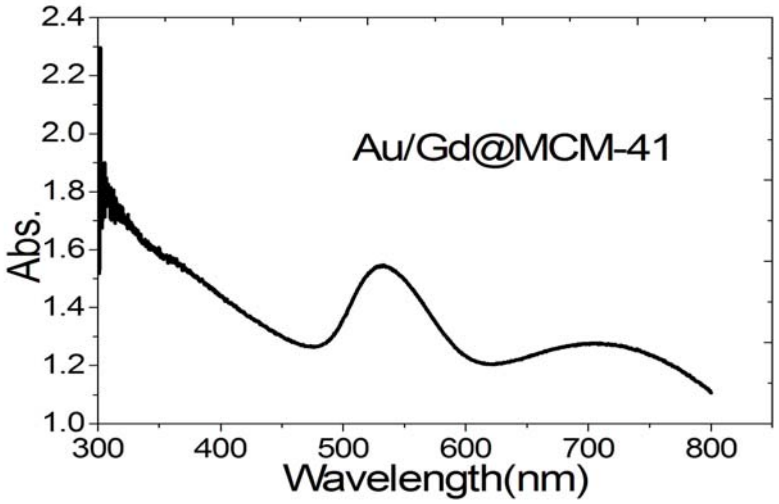

Figure S2 the steady- state absorption spectrum of Au/Gd@MCM-41.

2. **Raman Spectroscopy.** The measurements of Raman spectra analyses were performed on an Invia Raflex Raman spectrometer (Renishaw, Leica, UK). The light source is a Renishaw high power diode Laser and the wavelength is 785nm. The samples for Raman analysis were prepared by dispersing the nanocomposites in ethanol and drop on a cleaned silicon wafer with 10 s exposure time, and 100 s integration time.

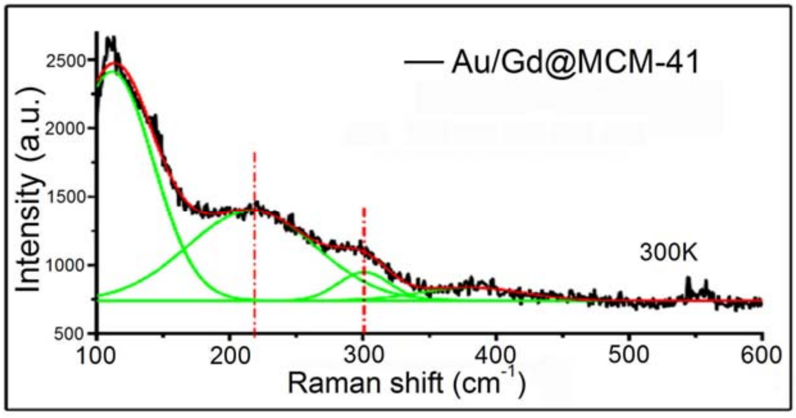

Figure S3 Raman spectra of Au/Gd@MCM-41 at room temperature. The experimental data were fitted to elucidate the peak position and full width of half-maxima of Raman peak. There are also four Raman peaks at 111.8, 216.4, 300.9 and 381.5cm^{-1}, respectively.

3. **In vitro Au/Gd@MCM-41 absorption data**. The transmission electron microscopy (TEM) images of CNE-2 cells which were cultured with Au/Gd@MCM-41 for 10h and 24h. Obviously, the Au/Gd@MCM-41 can be absorbed by the cells and well dispersed and distributed in CNE-2 cells.

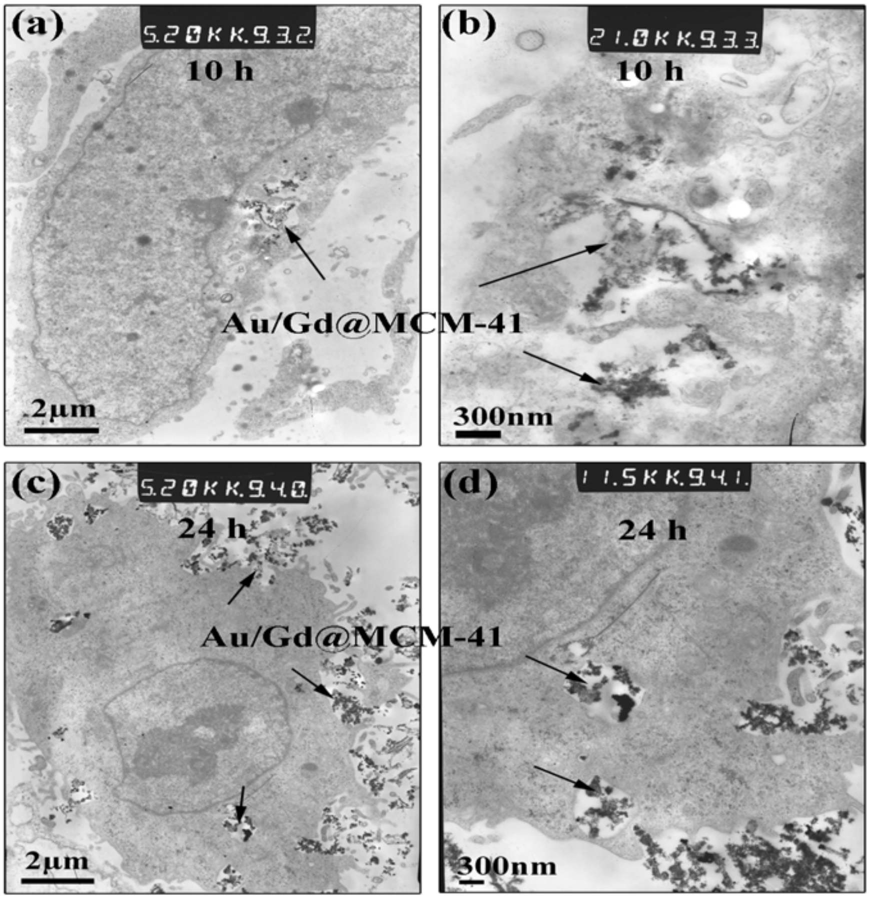

Figure S4 (a) The cells were cultured with Au/Gd@MCM-41 for 10 h. (b) High resolution version of (a). (c) The cells were cultured with Au/Gd@MCM-41 for 24 h. (d) High resolution version of (c).
